# Handgrip strength is associated with adverse outcomes in patients hospitalized for COVID-19-associated pneumonia

**DOI:** 10.1007/s11739-022-03060-3

**Published:** 2022-08-05

**Authors:** Giacomo Pucci, Marco D’Abbondanza, Rosa Curcio, Riccardo Alcidi, Tommaso Campanella, Lorenzo Chiatti, Vito Gandolfo, Vito Veca, Genni Casarola, Maria Comasia Leone, Rachele Rossi, Alessio Alberti, Leandro Sanesi, Massimiliano Cavallo, Gaetano Vaudo

**Affiliations:** 1 Department of Medicine and Surgery, University of Perugia, Unit of Internal Medicine, Terni University Hospital, Piazzale Tristano Di Joannuccio, 1, T05100 Terni, Italy; 2 COVID Unit, Terni University Hospital, Terni, Italy

**Keywords:** COVID-19, Respiratory failure, Handgrip strength, Sarcopenia, Muscular wasting, Obesity, Sarcopenic obesity

## Abstract

Handgrip strength (HGS), a simple tool for the evaluation of muscular strength, is independently associated with negative prognosis in many diseases. It is unknown whether HGS is prognostically relevant in COVID-19. We evaluated the ability of HGS to predict clinical outcomes in people with COVID-19-related pneumonia. 118 patients (66% men, 63 ± 12 years), consecutively hospitalized to the “Santa Maria” Terni University Hospital for COVID-19-related pneumonia and respiratory failure, underwent HGS measurement (Jamar hand-dynamometer) at ward admission. HGS was normalized to weight^2/3^ (nHGS) The main end-point was the first occurrence of death and/or endotracheal intubation at 14 days. Twenty-two patients reached the main end-point. In the Kaplan–Meyer analysis, the Log rank test showed significant differences between subjects with lower than mean HGS normalized to weight^2/3^ (nHGS) (< 1.32 kg/Kg^2/3^) vs subjects with higher than mean nHGS. (*p* = 0.03). In a Cox-proportional hazard model, nHGS inversely predicted the main end-point (hazard ratio, HR = 1.99 each 0.5 kg/Kg^2/3^ decrease, *p* = 0.03), independently from age, sex, body mass index, ratio of partial pressure arterial oxygen and fraction of inspired oxygen (PaO_2_/FiO_2_ ratio), hypertension, diabetes, estimated glomerular filtration rate and history of previous cardiovascular cardiovascular disease. These two latter also showed independent association with the main end-point (HR 1.30, *p* = 0.03 and 3.89, *p* < 0.01, respectively). In conclusion, nHGS measured at hospital admission, independently and inversely predicts the risk of poor outcomes in people with COVID-19-related pneumonia. The evaluation of HGS may be useful in early stratifying the risk of adverse prognosis in COVID-19.

## Introduction

The current impact of global outbreak of the coronavirus disease 2019 (COVID-19) on all-cause mortality and morbidity excess remains highly significant across all age spans [1, 2]. To improve risk stratification, set healthcare priorities and allow optimal allocation of resources, the evaluation of early prognostic markers of disease severity is of high importance.

Muscular involvement is a clinical manifestation of the multisystem COVID-19 syndrome. In COVID-19 patients, several reports described higher rates of muscle fatigue, weakness, and myalgias (often associated with increases in serum creatine phosphokinase (CPK), with an extent proportional to the gravity of the systemic inflammation [3–5]. This link is also suggested by the close correlation between serum CPK levels and inflammatory markers of disease such as c-reactive protein (CRP) [6].

Handgrip strength (HGS) is a simple, reproducible, and inexpensive tool for the evaluation of muscular strength in clinical practice through the measurement of the maximum static force that a hand can squeeze using a dynamometer. It is well acknowledged that HGS is a marker of global sarcopenia and muscular impairment and it is independently associated with increased risk of all-cause mortality in large-scale population-based studies [7, 8] as well as in critical illnesses [9]. Initial reports suggested that HGS was lower in COVID-19 patients, as compared to seronegative subjects [10]. Among COVID-19 hospitalized patients, HGS was consistently found to be closely correlated to indexes of disease severity even after accounting for age and gender differences [11]. However, the hypothesis that low HGS could predict future adverse clinical outcomes in patients hospitalized for severe COVID-19 has not been investigated to date.

The aim of the present study is to test the hypothesis that handgrip strength, a simple measure of global muscle strength, predicts short-term adverse outcomes in subjects with COVID-19-associated pneumonia independently from the potential effect of other clinical confounders.

## Methods

### Patients

All consecutive patients with COVID-19-related interstitial pneumonia and respiratory failure admitted to the “Santa Maria” Terni University Hospital, during the period between February and April 2021, except those who were directly admitted to intensive care units (ICU), were enrolled in the present study. The diagnosis of COVID-19 was based on positivity of viral RNA at the RT-PCR on the nasopharyngeal swab performed at Hospital admission, according to standardized procedures [12]. COVID-19-related interstitial pneumonia was diagnosed by radiological findings at the chest-X-ray or high-resolution computerized tomography (HRCT) scan. Respiratory failure was defined as standard partial oxygen pressure (PaO_2_) < 60 mmHg, evaluated through arterial blood gas analysis at hospital admission (Radiometer ABL90 FLEX) or need for oxygen support.

Exclusion criteria were: (1) absence of respiratory failure or any clinical or radiological sign of interstitial pneumonia; (2) moderate or advanced cognitive impairment or any other psychiatric disorder (e.g. delirium) resulting in an inability to execute simple verbal orders; (3) known neuromuscular degenerative or inflammatory diseases (such as amyotrophic lateral sclerosis, multiple sclerosis); (4) any other upper extremity musculoskeletal disease (e.g. polymyalgia rheumatica, rheumatoid arthritis, statin-related myopathy or known abnormal limitations of strength) or hand or wrist surgery within the previous 3 months; (5) advanced frailty (Clinical Frailty Scale [13] ≥ 7) due to cachexia or malnutrition.

All patients signed a written informed consent form to voluntarily participate in this study. A formal ethical approval was obtained from the local Ethics committee (acceptance protocol number: 908/2020). The study was conducted in accordance with the principles of the Declaration of Helsinki and good clinical practice guidelines of the International Conference of Harmonization (ICH GCP).

### Clinical evaluation

Clinical, demographic, and anthropometric parameters were evaluated at ward admission in all patients. Data about previous diseases and drug treatment were collected through direct medical interview, contact with members of the family or family doctors, and medical records. Previous cardiovascular disease was defined if a positive history of heart failure, coronary artery disease, stroke or transitory ischemic attack, aortic aneurysm or peripheral artery disease was present. A detailed clinical history related to SARS-CoV-2 infection was also taken, including potential sources of contagion, preceding symptoms, time from beginning of symptoms and hospital access. Weight and height were measured and body mass index (BMI) was calculated as weight (kg)/height^2^ with height expressed in (m^2^). Obesity was diagnosed if BMI was > 30 kg/m^2^. Complete laboratory assessment and arterial blood gas examination were performed at the time of hospital admission. Fraction of inspired oxygen (FiO_2_) was annotated and expressed as PaO_2_/FiO_2_ ratio. Estimated glomerular filtration rate (eGFR) was derived from the Chronic Kidney Disease Epidemiology Collaboration (CKD-EPI) creatinine equation.

All patients received an immediate bolus of dexamethasone (6 mg i.v.) according to current treatment guidelines for COVID-19-related respiratory failure [14]. Respiratory support was also immediately delivered according to respiratory failure severity through standard oxygen therapy (low flow nasal cannula oxygen system, reservoir system and high flow VenturiMask oxygen system), continuous positive airway pressure (cPAP), or bi-level non-invasive ventilation (NIV).

### Handgrip strength (HGS)

HGS measurement was performed at the time of the first clinical re-evaluation, occurring about 30 min after the beginning of immediate treatment procedures, upon achievement of a clinical steady state. HGS was measured through a calibrated Jamar hand dynamometer according to the American Society of Hand Therapists protocol [15]. All the patients were placed in seated position taking advantage, if needed, of electric adjustable beds. HGS measurement was taken with shoulder adducted, elbow flexed to 90 degrees, and forearm and wrist neutral. The operator placed the dynamometer in the patient’s dominant hand while lightly supporting the base of the dynamometer, he instructed the patient to squeeze as hard as possible for 2–3 s three times with 15 s of rest between measures on the same hand and encouraged the subject during the procedure. The test was performed by two trained medical staff (MDA, AA). The mean of three measurement at the dominant arm was used for the analysis.

Values of HGS were obtained in kilograms (Kg) and allometrically scaled to weight^2/3^ (normalized HGS, nHGS), according to previous literature, to exclude the confounding impact of obesity on handgrip strength [16]. Values of HGS were also compared, for each individual, with reference values derived from a cohort of 502,713 people from the general population, corresponding to the 50^th^ percentile according to sex, age, height and arm lateralization [15]. In a test cohort of 25 healthy individuals (mean age 42 ± 13 years, 45% women), the coefficient of variation of HGS measurements performed by the same medical staff within the study protocol was 5.3%.

### Outcome measures and sample size

The study design was prospective observational. The study hypothesis was that patients with higher than mean nHGS, compared to subjects with lower than mean nHGS were at increased risk of reaching the main endpoint. The main endpoint was the first occurrence of a composite outcome including death and/or need for endotracheal intubation within the acute phase of the disease, fixed at 14 days from the time of first HGS measurement. Those patients who were early discharged were contacted by phone by medical staff. Need for endotracheal intubation, based on the clinical course of the COVID-19-related respiratory failure, was judged on each patient together with the emergency physician/critical care consult team. This was mainly based on failure of non-invasive ventilation to maintain an adequate peripheral blood oxygenation or on the presence of clinical findings, e.g. fatigue of respiratory muscles. Potential contraindications to endotracheal intubation (e.g. upper way obstruction requiring surgical airway), or do-not-intubate (DNI) orders did not affect endpoint adjudication (e.g. if a patient was deemed to receive endotracheal intubation but was not intubated based on a DNI order, he was judged as reaching the main endpoint).

Assuming, from previous literature, an intubation rate of 20% in non-ICU patients with severe COVID-19 [17], it was estimated that enrollment of 114 patients would provide a ± 10% difference in the rate of composite endpoint occurring in patients with higher than mean nHGS compared to patients with lower than mean nHGS, with adequate power (70%) to rule out a Type I error with an alpha level of 0.05%.

### Statistical analysis

Descriptive statistics are presented as mean ± standard deviation or standard error where expressly reported. The assumption of satisfactory normal distribution was tested for all the examined variables by the Kolmogorov–Smirnov Z test. Pearson’s and Spearman’s correlation coefficients were used to assess the strength of correlation between variables. Differences between groups were tested with univariate comparisons and multivariate general linear models. The 14-day outcome of the two groups separated by the median value of the variable of interest (nHGS) were compared using Kaplan–Meier survival analysis. Univariate comparisons and multivariate Cox-proportional hazard models tested the association between nHGS and the main endpoint, with nHGS introduced as a continuous variable. In the multivariate model, age, sex, BMI, PaO_2_/FiO_2_ ratio, eGFR, and comorbidities such as hypertension, diabetes and previous cardiovascular (CV) disease were also included. The software used for statistical analysis was Statistical Package for Social Sciences (SPSS) version 26. A p value less than 0.05 was considered statistically significant.

## Results

**Fig. 1:**
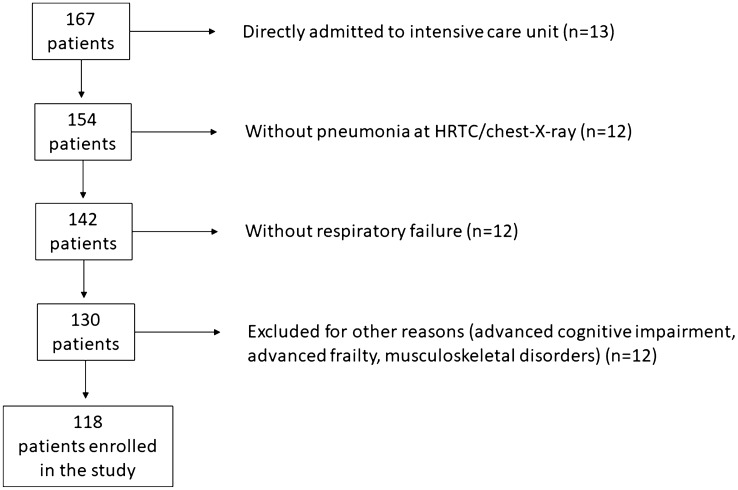
Trial profile of the study

From an unselected population of 154 patients hospitalized for COVID-19, 13 patients were directly admitted to ICU, 12 patients were excluded for absence of radiological signs of pneumonia at the chest-X-ray or HRCT scan, 12 patients were excluded for the absence of respiratory failure, and further 12 patients were excluded for the following reasons: 9 for advanced cognitive impairment, 2 for muscular disorders, 1 for cachexia and frailty. The remaining 118 patients (78 males, 40 females) were considered for the subsequent analysis. The trial profile is displayed in Fig. 1.

The main clinical and anthropometric characteristics are reported in Table 1. Patients were admitted to the hospital after 8 ± 6 days from the beginning of symptoms. Hypertension was present in the 46% of the study population, obesity was present in the 34%, 12% of patients reported a positive history of previous CV disease, 12% were diabetics. 13% of patients had an eGFR < 60 mL/min/1.73m^2^. After clinical stabilization, 15 patients (13%) were on treatment with NIV, 38 (32%) required cPAP, 65 (55%) were on conventional oxygen support. "Trial profile of thestudy".

**Table 1:** Clinical characteristics of the study population (*n* = 118)

Age, years	63 ± 12
Men, %	66
Height, cm	170 ± 10
Weight, Kg	85 ± 17
BMI, Kg/m^2^	29.1 ± 4.8
*Previous medical history*	
Hypertension, %	46
Obesity, %	34
T2DM, %	12
Current smoking, %	14
Previous CV disease, %	12
*Respiratory function*	
PaO_2_/FiO_2_ ratio	201 ± 77
NIV, *n* (%)	15 (13)
cPAP, *n* (%)	38 (32)
Conventional oxygen treatment, *n* (%)	65 (55)
*Laboratory assessment*	
eGFR < 60 mL/min/1,73m^2^, %	13
Serum CRP, mg/dL	9.1 ± 6.1
Serum ferritin, ng/L	806 ± 680
Serum LDH, U/L	329 ± 111
Absolute lymphocyte count/mm^3^	0.84 ± 0.41
Serum CPK, U/L	148 ± 232
Serum albumin, g/dL	3,3 ± 0.4
*Handgrip strength*	
Handgrip strength, Kg	27.1 ± 10
Handgrip strength/weight^2/3^, Kg/Kg^2/3^	1.32 ± 0.5
Measured handgrip strength/reference handgrip strength, %	70 ± 16
Measured—reference handgrip strength*, Kg	− 8.1 ± 7

^*^Reference handgrip strength was derived in each individual according to sex, age, height and arm lateralization (see Ref. 47). *BMI* body mass index, *T2DM* type 2 diabetes mellitus, *COPD* chronic obstructive pulmonary disease, *CV* cardiovascular, *PaO2/FiO2* partial oxygen pressure/fraction of inspired oxygen, *NIV* non-invasive ventilation, *cPAP* continuous positive airway pressure, *eGFR* estimated glomerular filtration rate, *CRP* c-reactive protein, LDH: lactate dehydrogenase, *CPK* creatine phosphokinase

Mean HGS was 27.1 ± 10 kg, mean nHGS was 1.32 ± 0.5 kg/Kg^2/3^. HGS was, on average, 70 ± 16% of the expected reference values (− 8.1 ± 7 kg lower than reference HGS, *p* < 0.001). Lower HGS values compared to reference were found in 103 out of 118 patients (87%).

During the following 14 days from study admission, 22 patients reached the main end-point, which was endotracheal intubation in all patients. nHGS was lower in subjects reaching the main end-point than in the remaining population (1.21 ± 0.5 kg/Kg^2/3^ vs 1.49 ± 0.4 kg/Kg^2/3^, *p* = 0.01). These subjects were also more frequently diabetics and had more often a positive history of CV disease (Table 2). They also differed in terms of eGFR and PaO_2_/FiO_2_ ratio, but not in terms of age, sex, BMI, serum CRP, lactate dehydrogenase (LDH), CPK, ferritin levels, and absolute lymphocyte count at hospital admission. The percentage of measured/reference HGS was 26% lower in subjects reaching the main end-point vs those not reaching the main end-point (60% ± 16% vs 83% ± 15%, *p* < 0.01).

**Table 2:** Clinical findings in patients reaching vs not reaching the main endpoint according to the study protocol

	Patients reaching the main endpoint (*n* = 22)	Patients not reaching the main endpoint (*n* = 96)	*P*
Age, years	66.4 ± 12	62.4 ± 9	0.15
Sex M, %	68	67	0.91
BMI, Kg/m^2^	29.8 ± 4	29.0 ± 5	0.50
Hypertension, %	69	49	0.16
T2DM, %	31	7	< 0.01
Previous CV disease, %	31	6	< 0.01
eGFR, mL/min/1.73m^2^	75 ± 25	90 ± 25	0.01
Serum CRP, mg/dL	11.6 ± 4	8.9 ± 7	0.09
Serum LDH, U/L	357 ± 98	321 ± 115	0.21
Serum ferritin, ng/L	1054 ± 871	751 ± 620	0.16
Serum CPK, U/L	190 ± 265	145 ± 238	0.45
Absolute lymphocyte count/mm^3^	0.75 ± 0.27	0.82 ± 0.43	0.47
PaO_2_/FiO_2_ ratio	161 ± 58	208 ± 84	0.03
Handgrip strength/weight^2/3^, Kg/Kg^2/3^	1.21 ± 0.09	1.49 ± 0.06	0.01
Measured handgrip strength/reference handgrip strength*, %	60 ± 16	83 ± 15	< 0.01
Measured handgrip strength < reference handgrip strength*, %	100	85	0.04

^*^Reference handgrip strength was derived in each individual according to sex, age, height and arm lateralization (see Ref. 47). *BMI* body mass index, *T2DM* type 2 diabetes mellitus, *COPD* chronic obstructive pulmonary disease, *CV* cardiovascular, *PaO2/FiO2* partial oxygen pressure/fraction of inspired oxygen, *NIV* non-invasive ventilation, *cPAP* continuous positive airway pressure, *eGFR* estimated glomerular filtration rate,* CRP* c-reactive protein, *LDH* lactate dehydrogenase, *CPK* creatine phosphokinase

**Fig. 2:**
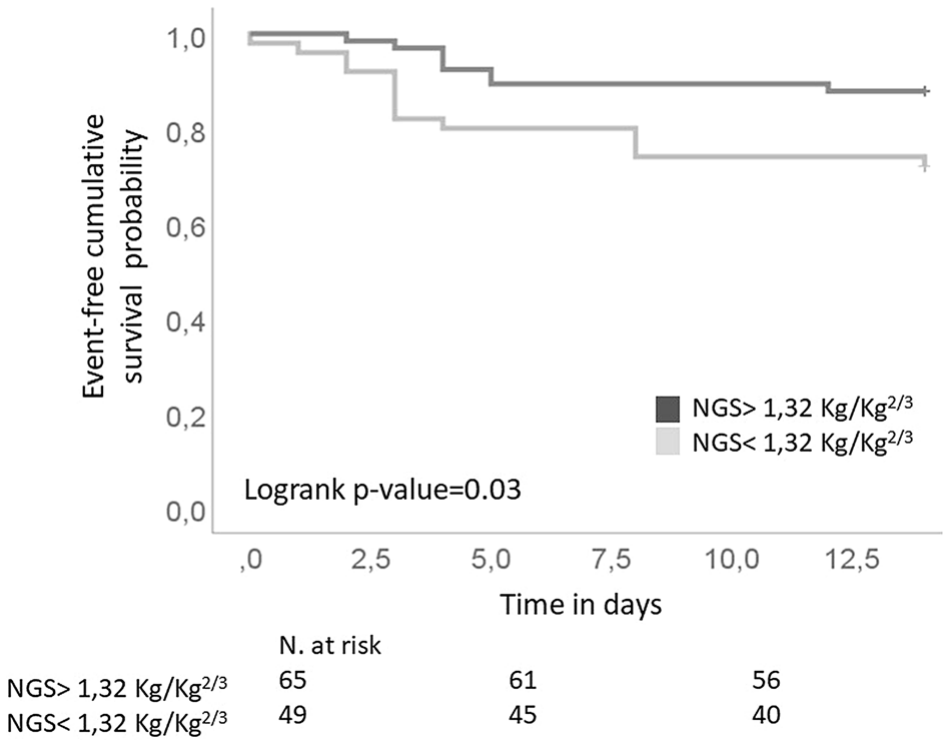
Kaplan–Meyer curve showing the probability of the main end-point stratified by mean normalized Handgrip Strength (nHGS), corresponding to 1.32 kg/Kg.^2/3^

The Kaplan–Meyer curve showed the probability of the main end-point stratified by mean nHGS, corresponding to 1.32 kg/Kg^2/3^ (Logrank *p* value = 0.03, Fig. 2). In a multivariate Cox-proportional hazard model, nHGS (adjusted hazard ratio -aHR- for each nHGS decrease of 0.5 kg/Kg^2/3^ = 1.99, 95% CI 1.01–3.91, *p* = 0.03) inversely predicted the occurrence of the main end-point independently from age, sex, BMI, PaO_2_/FiO_2_ ratio, eGFR, and comorbidities such as hypertension, diabetes and previous CV disease. eGFR (aHR for each 10 mL/min/1.73 m^2^ decrease 1.30, 95% CI 1.03–1.64, *p* = 0.03) and history of previous CV disease (aHR 3.89, 95% CI 1.14–13.24, *p* < 0.01) were two other independent predictors of adverse outcome in the study population (Table 3).

**Table 3:** Univariate and multivariate Cox-proportional hazard models exploring the role of independent predictors for the time to reach the main outcome (first occurrence of death and/or endotracheal intubation) during the acute phase of the disease

	Univariate				Multivariate			
	HR	Lower	Upper	*p* value	HR	Lower	Upper	*p* value
nHGS (each 0.5 kg/Kg^2/3^ decrease)	2.47	1.06	5.79	0.03	1.99	1.01	3.91	0.03
Age (years)	1.28	0.88	1.86	0.19	–	–	–	–
Male sex	1.02	0.42	2.51	0.95	–	–	–	–
BMI (Kg/m^2^)	1.03	0.95	1.11	0.48	–	–	–	–
Previous CV disease	6.63	2.47	17.85	< 0.01	3.89	1.14	13.24	< 0.01
eGFR (each 10 mL/min/1.73m^2^ decrease)	1.32	1.08	1.71	0.01	1.30	1.03	1.64	0.03
Hypertension	2.06	0.72	5.95	0.18	–	–	–	–
Diabetes mellitus	4.63	1.60	13.4	< 0.01	–	–	–	–
PaO2/FiO2 ratio (each 10 unit decrease)	1.08	1.01	1.17	0.035	–	–	–	–

All the listed variables were included in the multivariate model. The adjusted hazard ratio of variables independently associated with the main outcome were reported in terms of hazard ratio (HR), 95% confidence intervals (lower, upper), and *p* value. HR of variables not significantly associated with the main outcome in the multivariate model were not reported. *nHGS* handgrip strength normalized to weight^2/3^, *eGFR* estimated glomerular filtration rate, *BMI* body mass index. *CV* cardiovascular, *PaO2/FiO2* partial oxygen pressure/fraction of inspired oxygen

## Discussion

Results from our study showed that in a cohort of patients hospitalized in non-ICU wards for COVID-19-related interstitial pneumonia and respiratory failure, HGS normalized to weight ^2/3^ inversely predicted the risk of future short-term adverse clinical events, such as endotracheal intubation. Such risk was independent from the presence of confounders such as age, sex, BMI, other comorbidities, and indexes related to respiratory failure. Given the remarkable negative impact of endotracheal intubation on overall survival in severe COVID-19 [18], our observations raise the hypothesis that in patients with COVID-19-related respiratory failure, measurement of HGS could represent an easy, fast and portable tool to early identify patients at higher risk of complications and adverse prognosis and, therefore, might help in stratifying the risk of future events beyond classical risk factors. Indeed, in our population, each decrease in 10 kg/Kg^2/3^ upper arm muscle strength was associated to a nearly twofold risk of endotracheal intubation. We also found, in line with previous reports [19], that a positive history of CV diseases and reduced eGFR also independently predicted adverse clinical outcomes in COVID-19-related pneumonia.

The relationship between HGS, SARS-CoV-2 infection and severity of COVID-19 has been the object of previous research. In a population of older adults undergoing longitudinal HGS assessment, incident SARS-CoV-2 infection was independently associated with a more marked age-related HGS decline in comparison to seronegative individuals [20]. In the presence of SARS-CoV-2 infection, low HGS was also associated with an increased risk of hospitalization for COVID-19 [21]. Finally, among COVID-19 hospitalized patients, HGS was consistently found to be closely correlated to indexes of disease severity even after accounting for age and gender differences [11]. All these observations are concordant in generating the hypothesis that SARS-CoV-2 infection and related COVID-19 could impair muscular strength. However, to the best of our knowledge, our study was the first, to date, to unveil the potential of HGS to predict future adverse clinical outcomes in the hospital setting. This could be of high importance, especially in low-resource settings, to improve risk stratification at the time of first clinical stabilization after hospital admission, to set healthcare priorities and rationalize the allocation of medical resources.

It is well acknowledged that HGS is marker of global sarcopenia and muscular impairment and its association with increased risk of all-cause mortality has been previously described in population-based studies [7, 8]. However, the pathophysiological mechanisms through which low HGS could affect short-term clinical outcomes in COVID-19-associated pneumonia remain to be demonstrated. Indeed, a low HGS, evaluated at Hospital admission, could not only reflect the impact of COVID-19 but also be influenced by pre-existing chronic conditions known to affect global muscular impairment and sarcopenic status. Therefore, a certain loss of muscular strength might already have been supposed in people with chronic diseases [22, 23]. The evidence, however, that a very high number of patients showed HGS values far below expected reference values [15] also suggest that COVID-19 and related consequences might have an independent role in determining reduced HGS in the early phases of the disease. Our study was not designed to distinguish the impact of pre-existing sarcopenia and muscle wasting on future clinical outcomes, therefore, further studies are needed to better describe this aspect. Nevertheless, our results demonstrate the ability of HGS as a useful and inexpensive tool to predict risk of future adverse outcomes in patients hospitalized for COVID-19 independently from the etiology of muscular impairment.

In obese individuals, the evaluation of sarcopenia through the measurement of muscular strength is often masked by larger muscle size and for this reason, it frequently goes unnoticed [24, 25]. This is of importance, given that obesity is highly prevalent among patients with severe COVID-19, and it is also an independent risk factor of adverse outcomes [26]. Of note, in our population, we found reduced HGS values even after accounting for allometrical normalization of HGS for weight, and also that the association with nHGS with adverse outcomes remained significant in the multivariate model even after considering the potential impact of BMI on the outcome measure.

Other limitations should be acknowledged. Results from our study should be conceived only as hypothesis-generating and need further confirmation in large-scale studies. The relatively small sample size translates into a lack of adequate power to correctly assess if HGS has discriminative ability or added value in terms of net reclassification improvement when combined to classical severity scores currently proposed in COVID-19-related respiratory failure to stratify the risk [27]. The evaluation of HGS was performed only at study admission and results do not take into account of the potential impact of HGS changes during the subacute phase of COVID-19, during early phases of hospital stay, and also in response to targeted treatments. Other measures of muscular mass, physical performance, or indirect signs of muscle loss as indicators of sarcopenic status were not accounted in the present analysis, therefore, their potential impact on prognosis cannot be compared with that of HGS. Finally, given the lack information about previous nutritional status and degree of habitual physical exercise, their relative contribution on measured HGS and on subsequent clinically relevant events could not be precisely assessed.

Our results are in favor of the hypothesis that screening of wasting disorders such as sarcopenia and muscular impairment in people hospitalized for COVID-19, through easy to perform, highly reproducible and clinically relevant tools such as HGS, may be useful to improve risk prediction. Our results could also inspire further interventional studies targeted to evaluate the potential role of treatment with high-protein diets and nutritional supplements during hospital stay in mitigating muscle energy loss and possibly to reduce the incidence of adverse clinical outcomes in patients with severe COVID-19-related respiratory failure.
